# Insights into Antimalarial Activity of *N*-Phenyl-Substituted Cinnamanilides

**DOI:** 10.3390/molecules27227799

**Published:** 2022-11-12

**Authors:** Jiri Kos, Gilles Degotte, Dominika Pindjakova, Tomas Strharsky, Timotej Jankech, Tomas Gonec, Pierre Francotte, Michel Frederich, Josef Jampilek

**Affiliations:** 1 Department of Biochemistry, Faculty of Medicine, Masaryk University, Kamenice 5, 62500 Brno, Czech Republic; 2 Department of Analytical Chemistry, Faculty of Natural Sciences, Comenius University, Ilkovicova 6, 84215 Bratislava, Slovakia; 3 I2BM, Department of Molecular Chemistry, University Grenoble-Alpes, Rue de la Chimie 570, 38610 Gieres, France; 4 Department of Chemical Drugs, Faculty of Pharmacy, Masaryk University, Palackeho 1946/1, 61200 Brno, Czech Republic; 5 Laboratory of Medicinal Chemistry, CIRM, Department of Pharmacy, University of Liege, Avenue Hippocrate 15, 4000 Liege, Belgium; 6 Laboratory of Pharmacognosy, CIRM, Department of Pharmacy, University of Liege, Avenue Hippocrate 15, 4000 Liege, Belgium; 7 Department of Chemical Biology, Faculty of Science, Palacky University Olomouc, Slechtitelu 27, 78371 Olomouc, Czech Republic

**Keywords:** cinnamanilides, antiplasmodial activity, *Plasmodium*, structure-activity relationships

## Abstract

Due to the urgent need of innovation in the antimalarial therapeutic arsenal, a series of thirty-seven ring-substituted *N*-arylcinnamanilides prepared by microwave-assisted synthesis were subjected to primary screening against the chloroquine-sensitive strain of *P. falciparum* 3D7/MRA-102. The lipophilicity of all compounds was experimentally determined as the logarithm of the capacity factor *k*, and these data were subsequently used in the discussion of structure-activity relationships. Among the screened compounds, fourteen derivatives exhibited IC_50_ from 0.58 to 31 µM, whereas (2*E*)-*N*-(4-bromo-2-chlorophenyl)-3-phenylprop-2-enamide (**24**) was the most effective agent (IC_50_ = 0.58 µM). In addition, (2*E*)-*N*-[2,6-dibromo-4-(trifluoromethyl)- phenyl]-3-phenylprop-2-enamide (**36**), (2*E*)-*N*-[4-nitro-3-(trifluoromethyl)phenyl]-3-phenylprop- 2-enamide (**18**), (2*E*)-*N*-(2-bromo-5-fluorophenyl)-3-phenylprop-2-enamide (**23**), and (2*E*)-3-phenyl-*N*-(3,4,5-trichlorophenyl)prop-2-enamide (**33**) demonstrated efficacy in the IC_50_ range from 2.0 to 4.3 µM, comparable to the clinically used standard chloroquine. The results of a cell viability screening performed using THP1-Blue™ NF-κB cells showed that none of these highly active compounds displayed any significant cytotoxic effect up to 20 μM, which makes them promising *Plasmodium* selective substances for further investigations.

## 1. Introduction

Malaria is one of the most important infections and a life-threatening disease that is caused by parasites *Plasmodium falciparum*, *P. vivax*, *P. ovale*, *P. knowlesi*, and *P. malariae*, which are transmitted by infected female *Anopheles* mosquitoes. *P. falciparum* is the deadliest and the most widespread in Africa, while *P. vivax* is dominant outside Africa. After being bitten by an infectious mosquito and infected with the parasite, the patient develops flu-like symptoms (fever, headache, and chills) after 10 days, making it difficult to associate with malaria. However, if the infection caused by *P. falciparum* is untreated, it can rapidly progress to severe illness and death within 24 h. Severe health issues include anemia, hypoglycemia, pulmonary edema, kidney damage, jaundice, acidosis, coma, and death. The risk of death increases after reaching only 2% of infected erythrocytes in the blood [1]. Clinically used antimalarial drugs include quinine, chloroquine, primaquine and other aminoquinoline-based derivatives, sulfonamides, pyrimethamine, artemisinin and its analogues/prodrugs, as well as some antibiotics [2,3,4].

In 2020, there were an estimated 241 million cases of malaria worldwide, and the estimated number of malaria deaths stood at 627,000, after years of constant decrease [5]. A major cause of concern is the emergence of resistant and multidrug-resistant strains in Africa, including isolates resistant to chloroquine and artemisinin [6,7]. Another undesirable fact is that, due to climate change, the parasite and its vectors could spread to areas where they were eradicated or not present before [8,9]. In 2020, nearly half of the world’s population was at risk of malaria [5]. To face this undesirable situation, there is an urgent need for innovations in the therapeutic arsenal and the search for new antimalarial drugs.

In addition to analogues of synthetic antimalarials, scientists are turning to natural substances [2,10,11,12,13], where cinnamic acid and its derivatives, which have been used in traditional medicine for centuries and are known for a wide range of biological effects, represent one of the most effective lead compounds in drug design [14,15]. However, their antiplasmodial activities are poorly described. For example, only cinnamoyl-4-animoquinoline hybrids effective against the chloroquine-resistant *P. falciparum* strain W2 [16,17] or multi-substituted cinnamanilides effective against the *P. falciparum* strain Dd2 [18] have been reported.

Through the simplification of the structure of ring-substituted naphthalenes (e.g., [19,20,21]), we produced the (*E*)-prop-1-en-1-ylbenzene scaffold and thus, synthetized variously substituted anilides derived from cinnamic acids [22,23,24]. This basic series displayed antistaphylococcal, antifungal, and antituberculotic activity. Such a simple, diversely substituted, and easy-to-prepare library of anilides has not yet been evaluated against *Plasmodia*. Thus, this is a pilot screening of these compounds, which is logically performed on a *P. falciparum*-sensitive strain and provides preliminary data. It was typical for naphthalene-carboxanilides, models of these cinnamanilides, that an antimycobacterial effect was combined with the ability to inhibit photosynthetic electron transport in chloroplasts. It was shown that these compounds act via inhibition of photosystem II in plastoquinone Q_B_ in the thylakoid membrane, and, at the same time, are able to inhibit the mycobacterial proton pump F_0_F_1_H^+^ATPase, i.e., to inhibit the respiratory chain of mycobacteria; thus, they affect similar enzymatic systems in completely different biological entities [19,20,25,26,27]. Similarly, it has been shown that some antitubercular agents may have associated activity against *Plasmodia* through the modulation of structurally similar targets [28,29,30,31,32]. Thus, in addition to the study of the antimalarial potential of the basic series of cinnamanilides (substituted only on the anilide ring), finding a possible correlation between antiplasmodial and antitubercular activities became another goal.

## 2. Results and Discussion

### 2.1. Chemistry and Physicochemical Properties

**Scheme 1:**
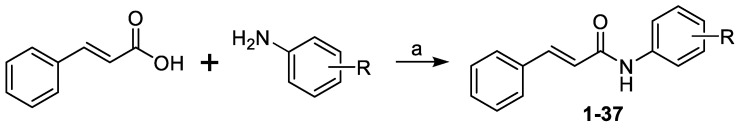
Synthesis of (2*E*)-*N*-aryl-3-phenylprop-2-enamides **1**–**37**. *Reagents and conditions:* (a) PCl_3_, chlorobenzene, MW, 20 min for **1**–**7**, **9**–**12**, **14**–**16**, **20**, **22**, **23**, **28** [22,33], or 30 min for **8**, **13**, **17**–**19**, **21**, **24**–**27**, **29**–**37** [23].

The synthesis of anilides **1**–**37** is shown in Scheme 1. For a one-step “click” preparation, a microwave-assisted synthesis was used, where the carboxyl group of cinnamic acid was first activated in dry chlorobenzene with phosphorus trichloride. The resulting acyl chloride subsequently provided the target anilide by means of the appropriate aniline. The synthesis and characterization of all investigated compounds is described in detail in [22,23,33].

Among the important parameters characterizing the physicochemical properties of the compounds, we studied the lipophilicity and electronic relations in the molecule. Electronic σ parameters of the whole substituted anilide ring, characterizing the ability to withdraw or donate electrons to the molecule system, were predicted by ACD/Percepta software and are listed in Table 1. The lipophilicity was determined using RP-HPLC as the logarithm of capacity factor *k*. The retention times of individual compounds were determined under isocratic conditions, with methanol as an organic modifier in the mobile phase using end-capped non-polar C18 stationary RP columns. In addition, the lipophilicity expressed as log *P* and Clog *P* was calculated using two commercially available programs: ACD/Percepta ver. 2012, and ChemBioDraw Ultra 13.0. All results are shown in Table 1.

**Figure 1:**
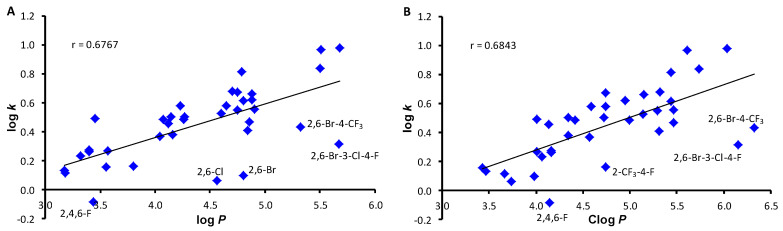
Comparison of experimentally found log *k* values of ring-substituted *N*-arylcinnamanilides **1**–**37** with log *P* calculated using ACD/Percepta (**A**) and Clog *P* calculated using ChemBio DrawUltra (**B**).

A correlation was made between the experimental values of log *k* and the predicted values of log *P*/Clog *P* (Figure 1). As demonstrated by the correlation coefficients in Figure 1a,b; *r* = 0.6767 and 0.6843 (*n* = 37), respectively; these correlations showed a rather limited agreement. Derivatives with large deviations are shown in individual graphs. Thus, it can be concluded that none of the selected software takes into account the interactions of molecules with the aqueous environment. These observations for the whole series contradict the previous finding where only mono-, dimethyl-, dichloro-, and dibromo-substituted derivatives were compared and the correlation coefficients r = 0.9774 for the dependence of log *k* on log *P* and r = 0.9513 for the correlation log *k* with Clog *P* were found [22].

**Figure 2:**
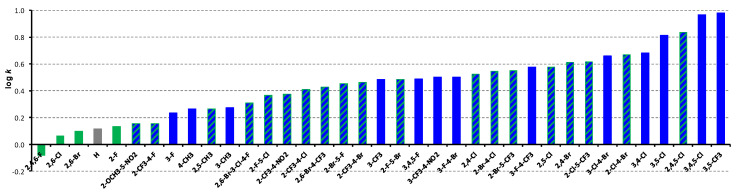
Order of individual derivatives arranged according to increasing log *k* values. (Grey = unsubstituted **1**, green = *ortho*-substituted derivatives, blue = *meta/para*-substituted derivatives, green/blue hatched = derivatives with *meta*/*para-* and *ortho*-substitutions.)

According to the experimental log *k* values, (2*E*)-3-phenyl-*N*-(2,4,6-trifluorophenyl)- prop-2-enamide (**32**) is the least lipophilic, followed by derivatives **10** (R = 2,6-Cl) and **11** (R = 2,6-Br). Conversely, the most lipophilic compounds are (2*E*)-3-phenyl-*N*- (3,4,5-trichlorophenyl)prop-2-enamide (**35**) and (2*E*)-*N*-[3,5-bis(trifluoromethyl)phenyl]- 3-phenylprop-2-enamide (**15**). It can be stated that substitution of both *ortho* positions (or at least one *ortho* position) significantly decreases lipophilicity, while substitution of *meta* and *para* positions increases lipophilicity. The highest lipophilicity is provided by disubstitution of both *meta* positions, followed by the disubstitution of the *meta* and *para* positions of the anilide. Tri- or tetrasubstitutions (e.g., compounds **36**, **37**) do not automatically provide the highest log *k* value. Interesting results are provided by various combinations of halogens at different positions, as well as by the three derivatives substituted with an NO_2_ group (**16**–**18**), as shown in Figure 2.

### 2.2. In Vitro Antiplasmodial and Toxicity Evaluation

The in vitro preliminary screening of the library of 37 compounds on a chloroquine-sensitive strain of *P. falciparum* 3D7/MRA-102 highlighted that 14 compounds possessed an IC_50_ < 35 µM and could be considered as active antiplasmodial agents. Among these 14 structures, (2*E*)-*N*-(4-bromo-2-chlorophenyl)-3-phenylprop-2-enamide (**24**) was the most active with IC_50_ = 0.58 µM, followed by (2*E*)-*N*-[2,6-dibromo- 4-(trifluoromethyl)phenyl]-3-phenylprop-2-enamide (**36**), (2*E*)-*N*-[4-nitro-3-(trifluoro- methyl)phenyl]-3-phenylprop-2-enamide (**18**), (2*E*)-*N*-(2-bromo-5-fluorophenyl)- 3-phenylprop-2-enamide (**23**), and (2*E*)-3-phenyl-*N*-(3,4,5-trichlorophenyl)prop- 2-enamide (**33**), with IC_50_ ranging from 2.0 to 4.3 µM. An overview of the activities is presented in Table 1.

It should be noted that all the compounds were previously evaluated for their cytotoxic potential on the human monocytic leukemia cell line THP1-Blue™ NF-κB [23,33]. Only compounds **6** (R = 3-CF_3_, IC_50_ = 11.60 ± 1.13 µM), **11** (R = 3,4-Cl, IC_50_ = 6.28 ± 2.32 µM), **12** (R = 3,5-Cl, IC_50_ = 2.43 ± 1.06 µM), **15** (R = 3,5-CF_3_, IC_50_ = 2.17 ± 1.19 µM), and **25** (R = 3-Cl-4-Br, IC_50_ = 6.5 ± 1.0 µM) [23,33] had a cytotoxic effect. It follows that all the highly anti-*Plasmodium* effective compounds demonstrated insignificant cytotoxicity (IC_50_ > 20 µM), making them promising for further investigations because of their apparent selectivity for the parasite.

Table 1 also contains the minimum inhibitory concentration (MIC) values against *Mycobacterium tuberculosis* H37Ra/ATCC 25177. As mentioned above, it had already been reported that antitubercular activity could be correlated with antimalarial activity. In the case of these cinnamanilides, only the few following compounds displayed simultaneously antitubercular and antiplasmodial activity: (2*E*)-*N*-(3-methylphenyl)-3-phenyl- prop-2-enamide (**2**) (IC_50_ = 21.6 µM, MIC = 67.4 µM), (2*E*)-*N*-(3-fluorophenyl)-3-phenyl- prop-2-enamide (**5**) (IC_50_ = 30.8 µM, MIC = 66.5 µM), and derivative **15** (R = 3,5-CF_3_, IC_50_ = 6.5 µM, MIC = 22.3 µM). Consequently, it seemed that the two effects could not be correlated in this case since the most effective compounds against *P. falciparum* possessed no or limited antitubercular activity.

Finally, the cinnamanilides were evaluated for their hemolytic potential to confirm the observed in vitro anti*-Plasmodium* potency. Indeed, compounds inducing erythrocyte membrane disruption will cause a significant decrease in the parasitic growth because of its intracellular development. As a result, none of the tested products exhibited any hemolytic activities, confirming their antiplasmodial potential.

**Figure 3:**
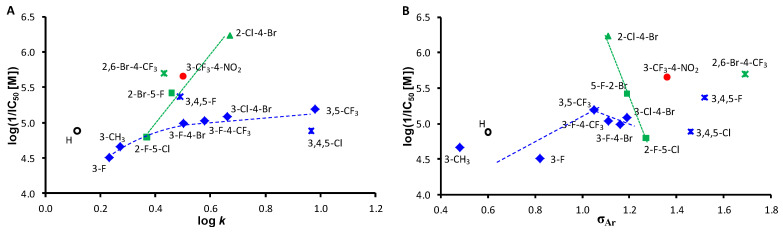
Relationships between in vitro activity against *P. falciparum* log(1/MIC [M]) and lipophilicity expressed as log *k* (**A**) and electronic σ parameters of substituted anilide ring (**B**) of studied compounds. (Black = unsubstituted derivative **1**, blue = *meta/para*-substituted derivatives, red = nitro-substituted derivative, green = *ortho*-substituted derivatives or derivatives with any *ortho*-substitution.)

Consequently, structure-activity relationships (SAR) were studied in a group of active derivatives. The dependences of the activity of the compounds against *P. falciparum* expressed as log(1/IC_50_ [M]) on lipophilicity expressed as log *k* are presented in Figure 3a. Although the correlation between antitubercular and antiplasmodial activity has not been confirmed, very similar SAR to those discussed by Pospisilova et al. have been identified [22]. In fact, two different dependences in relation to the position and the type of substituents can be observed. Based on Figure 3a, it can be stated that compounds substituted in positions C_(3)_′, C_(3,4)_′, or C_(3,5)_′ displayed an increasing trend of activity with the lipophilicity increasing up to compound **21** (R = 3-F-4-Br, log *k* ca. 0.5), after which the activity increases insignificantly. A similar correlation between antiplasmodial activity and lipophilicity has already been reported for 3,4-dihydroxycinnamic (caffeic) acid. This suggested that the compounds possessing a higher lipophilicity value could more easily enter inside the erythrocytes, reaching higher intracellular concentrations to exert their pharmacological effects. It has also been demonstrated that this potency increment seemed to be limited to an optimum lipophilicity value after which the activity stagnates or even decreases because of a higher affinity of the product for the cell membrane compared to the cytosol [34,35,36].

The second correlation, a linear and steeply increasing trend, can be found for the compounds substituted in *ortho* positions, i.e., C_(2,5)_′ and C_(2,4)_′. The activity increases from log *k* = 0.36 (comp. **19**, R = 2-F-5-Cl) to the most potent derivative **23** (log *k* = 0.67). The simultaneous substitution of the *para*- and *ortho*-substitution seems to be highly important, e.g., compound **14** (R = 2,6-Br) expressed an IC_50_ > 35 µM compared to compound **36** (R = 2,6-Br-4-CF_3_) with an IC_50_ = 2.0 µM. The activity of derivative **18** (R = 3-CF_3_-4-NO_2_) is specific and must be attributed only to this combination, because compound **17**, where the CF_3_ group is shifted to the C_(2)_′ position, already demonstrated only IC_50_ > 35 µM, similar to the combination R = 2-OCH_3_-5-NO_2_ (**16**).

It should also be noted that the antiplasmodial activity of the discussed cinnamanilides is also dependent on the electronic σ parameters, as shown in Figure 3b. A bilinear trend with an optimum of σ_Ar_ ca. 1.0 (comp. **15**, R = 3,5-CF_3_) can be found for compounds substituted at the C_(3)_′, C_(3,4)_′, and C_(3,5)_′ positions. The second trend of a steep decrease in activity with an increase in the electron-withdrawing properties of the anilide substituents is seen once again for the *ortho*-substituted derivatives (**23** (R = 2-Cl-4-Br) with σ = 1.1). Again, nitro-substituted derivative **18** with σ_Ar_ ca. 1.4 is specific.

In summary, the position of the substituents is crucial for the activity, while disubstitution of the *ortho* and *para* positions by combination of halogens (not of the same atoms, see inactive **8** (R = 2,4-Cl), **13** (R = 2,4-Br)) is advantageous. If the compound is substituted with CF_3_, the *meta* or *para* position seems to be preferred. This combination of substituents on the anilide ring ensures a suitable interval of lipophilicity in the range of log *k* 0.5–1 and electron-withdrawing properties characterized by σ_Ar_ in the interval from 1.1 to 1.7.

The molecules described in [18,36,37] may be considered structurally related to the compounds discussed herein. In the caffeic acid esters and esters of derivatives of chlorogenic acid described by Gilles et al. [36] and Alson et al. [37], both the ester and acid parts of the molecules were changed. Small lipophilic molecules were active within the described series. The introduction of a substituted sugar residue (chlorogenic acid derivatives) resulted in a significant reduction of plasmodial activity. Both the starting acids and the ester part participated in the antiplasmodial activity of the described derivatives [36,37]. On the other hand, compounds reported by Wiesner et al. [18] contained the same amide part, and only the acid part was slightly changed. Chosen *N*-(4-amino-2-benzoylphenyl)-2-(4-methylphenyl)acetamide [38,39,40] was acylated with 3-phenylpropanoic or cinnamic acid, and these complex amides showed very similar antiplasmodial activity. Subsequently, the C_(4)_ position of the aromatic cinnamic core was substituted with halogens, methyl, and alkoxyls, while the found activities were insignificantly different from each other [18]. From these observations, it could be assumed that the intrinsic activity was not caused by the cinnamic acid residue, but by the amide part, i.e., *N*-(4-amino-2-benzoylphenyl)-2-(4-methylphenyl)acetamide, of the molecule, which was subsequently confirmed [41,42]. The anilides discussed in the current paper are closer to the molecules described in [36,37]; their in vitro anti*-Plasmodium* potency is based on the unique combination of the cinnamic acid fragment and suitably substituted aniline.

## 3. Materials and Methods

### 3.1. Chemistry

All the discussed *N*-arylcinnamanilides were previously prepared and characterized by Pospisilova et al. [22], Hosek et al. [33], and Kos et al. [23].

### 3.2. Lipophilicity Determination by RP-HPLC

The HPLC separation system Merck Hitachi LaChrom Elite^®^ equipped with a Merck Hitachi LaChrom Elite^®^ L-2455 Diode-Array Detector (Hitachi High Technologies America, San Jose, CA, USA) was used. A chromatographic column Symmetry^®^ C18 5 μm, 4.6 × 250 mm, Part No. W21751W016 (Waters Corp., Milford, MA, USA) was used. The HPLC separation process was monitored by the EZ CHROM Elite^®^ software ver. 3.2.0 (Hitachi High Technologies America). The total flow of the column was 1.0 mL/min, injection 10 μL, column temperature 40 °C, and sample temperature 10 °C. The detection wavelength 214 nm was chosen. A KI methanolic solution was used for the dead time (*t_d_*) determination. Retention times (*t_r_*) were measured in minutes. Isocratic elution by a mixture of Metanol Chromasolv^TM^ (Honeywell, St. Louis, MO, USA) (72%) and H_2_O-HPLC Mili-Q grade (Labconco, Kansas City, MO, USA) (28%) as a mobile phase was used for the determination of capacity factor *k*. The capacity factors were calculated according to the formula *k* = (*t_r_* − *t_d_*)/*t_d_*, where *t_R_* is the retention time of the solute and *t_d_* is the dead time obtained using an unretained analyte. Each experiment was repeated three times. The calculated log *k* values of individual compounds are shown in Table 1.

### 3.3. In Vitro Antiplasmodial Activity

The following reagent was obtained through BEI Resources, NIAID, NIH: *Plasmodium falciparum*, strain 3D7, MRA-102, contributed by Daniel J. Carucci. Based on a modified procedure by Trager and Jensen [43], asexual erythrocytic stages of *Pf* were continuously maintained through in vitro culture. The chloroquine-sensitive strain is cultured thanks to human red blood cells (A+) and a culture medium mainly composed of RPMI 1640 (Gibco, Fisher Scientific, Loughborough, UK) containing NaHCO_3_ (32 mM), HEPES (25 mM), and l-glutamine. The medium was supplemented with 1.76 g/L of glucose (Sigma-Aldrich, Machelen, Belgium), 44 mg/mL of hypoxanthine (Sigma-Aldrich, Machelen, Belgium), 100 mg/L of gentamycin (Gibco, Fisher Scientific, Loughborough, UK), and 10% human pooled serum (A+), as previously described [44]. Solutions of pure products were prepared in DMSO at 10 mg/mL. As DMSO is recognized as toxic for the parasites, the highest concentration of solvent to which they were exposed was 1%. Thus, primary solutions were diluted in a culture medium to reach 100 µg/mL in the first row of a 96-well plate. Therefore, each test sample was applied in a series of eight 2-fold dilutions and tested in triplicate. The assay was performed with 2% parasitaemia and 1% haematocrit [45]. After 48 h of incubation, plates were frozen at −20 °C for 12 h and parasite growth was quantified according to the methods described by Makler et al. [46]. Chloroquine (Sigma-Aldrich, Machelen, Belgium) was used as positive standards in all experiments, with initial concentrations at 100 ng/mL. Infected and uninfected red blood cells (RBC) were used as positive (100% growth) and negative controls (0% growth). Consequently, comparison between infected erythrocytes and samples allowed us to estimate the growth inhibition. IC_50_ values were calculated from linear regression. Due to the great number of compounds to test, a first experiment was performed (twice) with one concentration = 50 µg/mL. The molecules that did not reach 45% of inhibition at 50 µg/mL were discarded. The results are shown in Table 1.

### 3.4. In Vitro Hemolytic Activity

Hemolysis induction was evaluated for all the tested compounds based on a reported procedure [47]. Consequently, a 10% red blood cell suspension in PBS (*v*/*v*) (A+) was incubated with compounds in duplicate. The primary solutions were diluted in PBS to reach 100 μg/mL as the final concentration (DMSO < 1%). After agitation at room temperature for 1 h, the mixtures were centrifuged for 5 min at 2000 rpm, and 150 μL of supernatant was transferred to a 96-microwell plate. The absorbance was evaluated at 550 nm with a microplate reader (OD). The positive control was Triton X-100 1% (*v*/*v*) (corresponding to 100% lysis), and PBS was the negative control (corresponding to 0% lysis). The percentage of red blood cell lysis (*H*) was calculated as follows: *H* = (*OD*_550_ sample − *OD*_550_ PBS)/(*OD*_550_ Triton X-100 1% (*v*/*v*) − *OD*_550_ PBS) * 100. The hemolysis was considered as insignificant if it was lower than 1% of the total RBC.

## 4. Conclusions

Due to the global health threat that represents the emergence of resistant strains of *Plasmodium falciparum* on the African continent and the urgent need of innovations in the field of antimalarials, a set of 37 cinnamic acid anilides were preliminarily screened for their antiplasmodial potential. Indeed, similar scaffolds had already been reported [18,36,37] to significantly impede the intracellular parasite multiplication. Out of this focused library, 14 compounds exhibited a selective in vitro inhibitory effect on *P. falciparum* growth with IC_50_ quantified between 0.58 and 31 µM, (2*E*)-*N*-(4-bromo-2-chloro- phenyl)-3-phenylprop-2-enamide (**24**), as the most effective agent (IC_50_ = 0.58 µM), without any toxicity on THP1-Blue™ NF-κB cells.

Even though it was not possible to correlate the antitubercular effect of the products to their antiplasmodial activity [22,23], some structure-activity relationships were identified. Of note was the dependence of the potency to the lipophilicity of the molecules until an optimum log *k* value, suggesting an increased permeation rate, as already reported for other cinnamic acid derivatives [34,36,47]. In addition, the substitution pattern of the *N*-aryl seemed crucial for the anti-*Plasmodium* effect since the most efficient structures possessed at least two different halogen substituents. This suggests that the impact of the substitution on the electronic density of the cycle, as well as on the lipophilicity, is significant on the antiplasmodial effect.

As far as we know, the exact mechanism of action of cinnamic acid scaffolds on *P. falciparum* remains unknown, preventing the use of computational chemistry techniques to improve the reported submicromolar effect of compound **24**. However, similar scaffolds have been described as potent antiprotozoal agents on *Leishmania amazonensis* through the inhibition of arginase [48,49], involved in the pivotal polyamine pathway, and a similar enzyme has been reported in *P. falciparum* [50,51]. Therefore, one perspective could be the docking and the evaluation of the reported cinnamanilides against the *Pf* arginase, and the use of structure-based drug design to obtain more potent cinnamic acid derivatives before moving to in vivo experiments. Thus, the results obtained in this initial study with cinnamanilides will need to be supported by further testing on resistant *P. falciparum* strains, as well as by functional genomic and proteomic studies to reveal the mechanism of action.

## Figures and Tables

**Table 1:** Structures of ring-substituted (2*E*)-*N*-aryl-3-phenylprop-2-enamides **1**–**37**, experimentally determined values of lipophilicity log *k*, calculated values of log *P*/Clog *P*, electronic σ parameters, in vitro antiplasmodial activity (IC_50_ [µM] ± SD, *n* = 3) compared to chloroquine (CQ), and in vitro antitubercular activity (MIC [µM]) in comparison with isoniazid (INH).

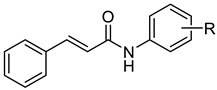							
No.	R	log *k*	log *P* ^1^	Clog *P* ^2^	σ_Ar_ ^1^	*Pf* IC_50_ [µM] ± SD	*Mtb* ^3^ MIC [μM]
**1**	H	0.1146	3.18	3.664	0.60	13.1 ± 5.9	286
**2**	3-CH_3_	0.2729	3.40	4.163	0.48	21.9 ± 6.3	67.4
**3**	4-CH_3_	0.2640	3.40	4.163	0.46	>35	134
**4**	2-F	0.1330	3.17	3.465	1.02	>35	265
**5**	3-F	0.2327	3.32	4.065	0.82	30.8 ± 2.4	66.3
**6**	3-CF_3_	0.4859	4.26	4.998	0.89	>35	27.5
**7**	2,5-CH_3_	0.2691	3.57	4.012	0.59	>35	254
**8**	2,4-Cl	0.5278	4.6	5.141	1.12	>35	>440
**9**	2,5-Cl	0.5799	4.65	4.588	1.22	>35	876
**10**	2,6-Cl	0.0632	4.56	3.738	1.33	>35	876
**11**	3,4-Cl	0.6821	4.70	5.318	1.19	>35	27.4
**12**	3,5-Cl	0.8155	4.79	5.438	1.11	>35	27.4
**13**	2,4-Br	0.6152	4.80	5.441	1.11	>35	>339
**14**	2,6-Br	0.0992	4.80	3.978	1.33	>35	167
**15**	3,5-CF_3_	0.9814	5.68	6.039	1.05	6.5 ± 3.6	22.3
**16**	2-OCH_3_-5-NO_2_	0.1581	3.55	3.433	1.32	>35	>859
**17**	2-CF_3_-4-NO_2_	0.3794	4.16	4.341	1.45	>35	>381
**18**	3-CF_3_-4-NO_2_	0.5004	4.27	4.341	1.36	2.2 ± 1.9	>381
**19**	2-F-5-Cl	0.3692	4.04	4.571	1.27	16.0 ± 4.2	>465
**20**	2-F-5-Br	0.4875	4.07	4.418	1.28	>35	199
**21**	3-F-4-Br	0.5025	4.14	4.721	1.16	10.3 ± 4.6	>414
**22**	3-F-4-CF_3_	0.5789	4.23	4.741	1.11	9.3 ± 5.3	>414
**23**	2-Br-5-F	0.4588	4.12	4.138	1.19	3.8 ± 0.61	199
**24**	2-Cl-4-Br	0.6710	4.75	4.738	1.11	0.58 ± 0.002	>766
**25**	3-Cl-4-Br	0.6611	4.88	5.151	1.19	8.2 ± 4.7	>383
**26**	2-Br-4-Cl	0.5476	4.75	5.291	1.12	>35	>383
**27**	2-Cl-5-CF_3_	0.6178	4.88	4.951	1.19	>35	785
**28**	2-Br-5-CF_3_	0.5561	4.90	5.461	1.19	>35	>348
**29**	2-CF_3_-4-F	0.1607	3.80	4.741	0.93	>35	>414
**30**	2-CF_3_-4-Cl	0.4113	4.84	5.311	1.06	>35	>394
**31**	2-CF_3_-4-Br	0.4683	4.86	5.461	1.05	>35	>348
**32**	2,4,6-F	−0.0831	3.44	4.144	1.46	>35	>462
**33**	3,4,5-F	0.4893	3.45	4.004	1.52	4.3 ± 2.4	>462
**34**	2,4,5-Cl	0.8373	5.5	5.734	1.56	>35	>395
**35**	3,4,5-Cl	0.9671	5.51	5.614	1.46	13.0 ± 3.8	>395
**36**	2,6-Br-4-CF_3_	0.4321	5.32	6.324	1.69	2.0 ± 1.9	>287
**37**	2,6-Br-3-Cl-4-F	0.3139	5.67	6.157	1.98	>35	>298
**CQ**	-	-	-	-	-	0.5 ± 0.2	-
**INH**	-	-	-	-	-	-	36.6

^1^ ACD/Percepta ver. 2012 (Advanced Chemistry Development. Inc., Toronto, ON, Canada, 2012); ^2^ ChemBioDrawUltra 13.0 (CambridgeSoft, PerkinElmer Inc., MA, USA). *Pf* = *Plasmodium falciparum* 3D7/MRA-102; *Mtb* = *Mycobacterium tuberculosis* H37Ra/ATCC 25177; ^3^ values taken from [22,23].

## Data Availability

The data presented in this study are available upon request from the corresponding authors.

## Sample Availability

Samples of the investigated compounds are available upon request from the corresponding authors.
